# Exploring Knowledge of Hypertension and Treatment Adherence in Hypertensive Patients From Lahore, Pakistan

**DOI:** 10.7759/cureus.81762

**Published:** 2025-04-05

**Authors:** Barak Waris, Nauman Ismat Butt, Ayesha Afzal, Muhammad Sohail Ajmal Ghoauri, Imania Khizar, Khalid Mahmood, Muhammad Atif Qureshi

**Affiliations:** 1 Medicine and Allied Health Sciences, Chaudhry Muhammad Akram Teaching and Research Hospital, Azra Naheed Medical College, Superior University, Lahore, PAK; 2 Internal Medicine/Rheumatology, Chaudhry Muhammad Akram Teaching and Research Hospital, Azra Naheed Medical College, Superior University, Lahore, PAK; 3 Medicine/Neurology, Bahawal Victoria Hospital, Quaid-e-Azam Medical College, Bahawalpur, PAK; 4 Internal Medicine and Medical Education, Chaudhry Muhammad Akram Teaching and Research Hospital, Azra Naheed Medical College, Superior University, Lahore, PAK

**Keywords:** blood pressure control, chronic kidney disease, diabetes mellitus, healthcare practices, hypertension, hypertension knowledge, ischemic heart disease, patient education, treatment adherence

## Abstract

Objective

This study aims to explore key factors influencing hypertension management, including demographic (e.g., age, gender, education level), clinical (e.g., presence of comorbid conditions), and behavioral influences (e.g., medication adherence, lifestyle choices such as diet and exercise). The study seeks to provide specific insights into the gaps in knowledge of hypertension and treatment adherence and identify potential barriers to effective treatment and lifestyle modifications.

Methods

This study was carried out in the Departments of Medicine and Cardiology at Chaudhary Muhammad Akram Teaching and Research Hospital, Azra Naheed Medical College, Superior University, Lahore, Pakistan, from November 2024 to February 2025. A total of 252 hypertensive patients, diagnosed for at least six months, aged ≥18 years, and of both genders, were enrolled using a nonprobability consecutive sampling technique after informed consent. Patients were excluded if they were unable to complete the questionnaire, had hypertensive emergencies, were critically ill or in the ICU, had an active myocardial infarction or stroke, were undergoing hemodialysis, or had a pre-existing psychiatric illness. A structured questionnaire was completed by the patients, collecting details including demographic and clinical information, the 12-item Hypertension Knowledge Test (HKT), and a 10-item domain regarding practices of hypertension control and treatment adherence. Both these questionnaires have been validated in previous studies. All data were entered into IBM SPSS Statistics for Windows, Version 23 (Released 2015; IBM Corp., Armonk, New York) for statistical analysis.

Results

The mean age was 47.6±12.9 years, with 133 (52.8%) participants aged ≥45 years. The majority were female (154, 61.1%). Of the 252 patients, 201 (79.8%) were married, 136 (54.0%) had ≤5 years of education, and 152 (60.3%) were employed. The mean duration of hypertension was 7.7±6.0 years, with 146 (57.9%) having hypertension for ≥6 years. Diabetes mellitus was present in 134 (53.2%), ischemic heart disease in 94 (37.3%), and chronic kidney disease in 28 (11.1%). A positive family history of hypertension was reported in 179 (71.0%). The mean HKT score was 7.1±2.0, with the majority (203, 80.6%) having poor knowledge. A significant statistical association of good knowledge was seen with younger age (p <0.001), being unmarried (p = 0.001), being educated (p <0.001), being employed (p <0.001), duration of hypertension ≤5 years (p = 0.039), absence of ischemic heart disease (p = 0.002), absence of chronic kidney disease (p = 0.024), and having a positive family history (p <0.001). No association was seen with gender (p = 0.336) or diabetes mellitus (p = 0.053). Regarding medication adherence, 26 (10.3%) always forget to take their medication, while 65 (25.8%) do so most of the time. In terms of healthcare management, 68 (27.0%) always make their next doctor’s appointment before leaving the office, while 99 (39.3%) do so most of the time. However, 21 (8.3%) have missed a scheduled appointment, and 54 (21.4%) do so most of the time.

Conclusion

This study underscores a significant gap in knowledge of hypertension and suboptimal adherence to treatment among hypertensive patients and highlights the need for educational programs and strategies to promote treatment adherence, especially among older patients and those with lower educational levels.

## Introduction

Hypertension, a leading cause of global morbidity and mortality, is defined by systolic blood pressure of 140 mmHg or higher or diastolic pressure of 90 mmHg or more, confirmed across multiple visits. It significantly raises the risk of cardiovascular and renal diseases, contributing to 51% of stroke-related deaths and 45% of heart disease fatalities worldwide [1]. The World Health Organization reports that nearly half of the population in both developing (47%) and developed (49%) countries are affected by hypertension, including both diagnosed cases and undiagnosed or prehypertensive individuals [2]. Hypertension is responsible for approximately 10.8 million deaths annually [3]. However, a lack of public awareness about hypertension management, such as the importance of lifestyle modifications (e.g., diet, exercise) and medication adherence, complicates efforts to control it effectively [4]. Poor treatment adherence, driven by factors like medication side effects, poor blood pressure control, and insufficient patient education, worsens health outcomes and increases healthcare costs [5]. 

Healthcare system-related factors, such as the availability of medications and effective doctor-patient communication, play a critical role in hypertension management. Limited access to essential medications, especially in resource-limited settings, can lead to gaps in treatment adherence and suboptimal blood pressure control. Additionally, poor communication between healthcare providers and patients may result in misunderstandings about the importance of medication adherence, lifestyle changes, and regular follow-ups. These system-related barriers, alongside individual factors, contribute significantly to the challenges in managing hypertension effectively. Addressing these issues is particularly crucial in Pakistan, where hypertension is highly prevalent [6,7]. Initiatives such as the National Action Plan for the Prevention and Control of Non-Communicable Diseases aim to improve knowledge and treatment adherence through public education and community interventions [8].

The rationale for conducting this study stems from the growing prevalence of hypertension and its associated complications, such as cardiovascular disease, stroke, and kidney failure, which pose significant public health challenges globally, including in Pakistan. Despite the availability of effective treatment options, hypertension remains underdiagnosed and poorly managed due to factors such as inadequate knowledge, poor adherence to treatment, and unhealthy lifestyle practices. In Pakistan, particularly in Lahore, limited research exists on patients' understanding of hypertension and their adherence to treatment protocols. Unlike previous studies that primarily focused on individual aspects of hypertension management, this research offers a more comprehensive approach by examining multiple influencing factors, including the role of doctor-patient communication and access to medications.

This study examines the combined impact of demographic, clinical, and healthcare system-related factors on knowledge of hypertension and treatment adherence in Lahore, Pakistan. Additionally, by focusing on a region with limited existing research on these issues, the study provides valuable insights that can inform targeted interventions and healthcare policies tailored to the local context. By addressing both individual and system-related barriers, it fills a gap in local research and offers new insights for targeted interventions. The findings can inform healthcare policies, guiding the development of educational programs and strategies to improve hypertension control and reduce health risks in the Pakistani population.

## Materials and methods

This cross-sectional research was carried out in the Departments of Medicine and Cardiology at Chaudhary Muhammad Akram Teaching and Research Hospital, Azra Naheed Medical College, Superior University, Lahore, Pakistan, from November 2024 to February 2025. This hospital was chosen for the study due to its status as a prominent healthcare facility, serving a diverse patient population with varying demographics, including those with chronic conditions like hypertension. As a referral center, it provides a wide range of specialized medical services, attracting patients from both urban and rural areas. Ethical clearance was granted by the Institutional Ethical Review Committee of Azra Naheed Medical College, Superior University, before data collection commenced. The study was conducted in accordance with the 1964 Declaration of Helsinki, as updated in 2000. Strict measures were implemented to maintain patient confidentiality and safeguard data privacy throughout the duration of the study. No identifiable patient information was included in the analysis, ensuring compliance with ethical research practices in healthcare.

Hypertension was defined as blood pressure exceeding 140/90 mm Hg on two or more separate occasions, either currently or in the past. Additionally, patients who were on antihypertensive medication were also considered hypertensive, regardless of their current blood pressure readings. This definition ensured that both diagnosed and treated individuals were included, capturing a comprehensive group of hypertensive patients for analysis. Diabetes mellitus was characterized by an HbA1c level greater than 6.5%, two random blood glucose readings ≥200 mg/dL, a fasting plasma glucose ≥126 mg/dL, a prior diagnosis of diabetes, or the use of antihyperglycemic medications. Chronic kidney disease was diagnosed based on an estimated glomerular filtration rate (eGFR) of less than 60 mL/min per 1.73 m² sustained for ≥3 months and ultrasonographic evidence of shrunken kidney size, thinning of the cortex, and increased cortical echogenicity. Ischemic heart disease was defined as a prior diagnosis of acute myocardial infarction or unstable angina.

The sample size for this study was calculated using the formula for cross-sectional studies, assuming a 95% confidence level, 5% margin of error, and an expected population proportion of 50%. The calculation yielded a required sample size of 252 patients, which was rounded up to account for potential non-responses and incomplete data. This sample size is sufficient to ensure the power and precision of the study findings. This conservative estimate ensures maximum variability and provides a more robust sample size, enhancing the precision of the study findings. Additionally, a nonresponse rate was anticipated and incorporated into the calculation to account for potential incomplete data or patient dropout, ensuring the final sample size of 252 patients would still provide adequate power to detect meaningful associations.

A total of 252 hypertensive patients, aged 18 years and older, of both genders, were enrolled in the study using a nonprobability consecutive sampling technique, after obtaining detailed informed consent from all participants. Patients were included if they had been diagnosed with hypertension for at least six months, ensuring a mix of newly diagnosed and long-standing cases. Patients were excluded from the study if they were unable to complete the questionnaire, had hypertensive emergencies, were critically ill or in the ICU, had an active myocardial infarction or stroke at the time of the study, were undergoing hemodialysis, or had a history of significant pre-existing psychiatric illness.

A structured questionnaire, consisting of three domains, was completed by the patients in the presence of a medical professional. The first domain collected demographic and clinical information, including age, gender, educational status, marital status, employment status, duration of hypertension, comorbid conditions, and family history of hypertension. The second domain comprised the hypertension knowledge test (HKT). The HKT is a 12-item questionnaire with true/false responses, yielding a maximum score of 12, adapted from Han et al. [9] and attached in the appendices. Modifications were made to ensure cultural and linguistic appropriateness for the study population to improve understanding and accessibility for patients, aiming to enhance the relevance of the test for the Pakistani population and ensure accurate assessment of hypertension knowledge. In this study, an HKT score of 0-8 was categorized as poor knowledge, while an HKT score of 9-12 was classified as good knowledge, using 75% of the total score as the cutoff. The third domain assessed practices related to hypertension control and treatment adherence, consisting of 10 questions, each rated on a Likert scale ranging from "never" to "all the time," and was adopted from Daniel et al. [10], with a Cronbach's alpha coefficient of 0.983.

All data were documented and entered into IBM SPSS Statistics for Windows, Version 23 (Released 2015; IBM Corp., Armonk, New York) for statistical analysis. For qualitative data, percentages and frequencies were computed, while means and standard deviations were determined for quantitative variables. Stratification was used to control for confounders and effect modifiers. Age and disease duration were grouped based on a 50% cumulative percentage to ensure balanced distribution. The education categories were defined as ≤5 years, 6-12 years, and ≥13 years to distinguish between individuals with limited (primary school), moderate (secondary school), and higher education levels, reflecting common educational attainment in this region. The chi-square test and Fisher's exact test were applied, with a p-value of <0.05 considered statistically significant.

## Results

The mean age of the patients was 47.6±12.9 years, with 133 (52.8%) aged ≥45 years, as shown in Table 1. The majority of the patients were female (154, 61.1%). Out of the 252 patients, 201 (79.8%) were married, 136 (54.0%) had ≤5 years of education, and 152 (60.3%) were unemployed, which included individuals who were retired, homemakers, or unable to work due to illness. The mean duration of disease (hypertension) was 7.7±6.0 years, with 146 (57.9%) having hypertension for ≥6 years. With regard to comorbid conditions, diabetes mellitus was present in 134 (53.2%), which was higher than ischemic heart disease in 94 (37.3%) and chronic kidney disease in 28 (11.1%) combined, as depicted in Table 1. A positive family history of hypertension was reported in 179 (71.0%). The mean HKT score was 7.1±2.0, with the majority of the participants having poor knowledge about hypertension (203, 80.6%).

**Table 1:** Demographic and clinical variables of the patients (n = 252) HKT: hypertension knowledge test.

Demographic and clinical variables	Category	Frequency, n (%)
Age	≤44 years	119 (47.2%)
	≥45 years	133 (52.8%)
Gender	Female	154 (61.1%)
	Male	98 (38.9%)
Marital status	Married	201 (79.8%)
	Unmarried	51 (20.2%)
Educational status	≤5 years	136 (54.0%)
	6-12 years	88 (34.9%)
	≥13 years	28 (11.1%)
Employment status	Employed	100 (39.7%)
	Unemployed	152 (60.3%)
Duration of disease (hypertension)	≤5 years	106 (42.1%)
	≥6 years	146 (57.9%)
Diabetes mellitus	Present	134 (53.2%)
	Absent	118 (46.8%)
Ischemic heart disease	Present	94 (37.3%)
	Absent	158 (62.7%)
Chronic kidney disease	Present	28 (11.1%)
	Absent	224 (88.9%)
Family history of hypertension	Present	179 (71.0%)
	Absent	73 (29.0%)
Hypertension knowledge according to the HKT score	Good (HKT score 9-12)	49 (19.4%)
	Poor (HKT score 0-8)	203 (80.6%)

A majority of patients (149, 59.1%) correctly identified that having a parent with high blood pressure (HBP) increases the likelihood of developing it, while 128 (50.8%) understood that HBP does not always present symptoms. As demonstrated in Table 2, 162 (64.3%) participants correctly rejected the notion that HBP is not life-threatening, and 164 (65.1%) accurately identified that blood pressure is considered high when it reaches or exceeds 140/90 mmHg. Additionally, 134 (53.2%) participants understood that being overweight significantly increases the risk of developing HBP, and 147 (58.3%) recognized that regular exercise can help lower blood pressure. As shown in Table 2, 172 (68.3%) correctly refuted the idea that alcohol lowers blood pressure, 168 (66.7%) correctly identified that pregnancy-related HBP requires follow-up care after delivery, while 146 (57.9%) understood that blood pressure does not typically decrease in cold weather. Finally, 126 (50.0%) participants acknowledged that Pakistanis consume much more salt than necessary.

**Table 2:** Response to individual questions of HKT (n = 252) HKT: hypertension knowledge test.

HKT statement	Correct answer	Frequency, n (%)
If your mother or father has high blood pressure (HBP), your chance of getting it is higher.	True	149 (59.1%)
Young adults don't get HBP.	False	156 (61.9%)
HBP always has symptoms.	False	128 (50.8%)
HBP is not life-threatening.	False	162 (64.3%)
Blood pressure (BP) is high when it's at or over 140/90 mmHg.	True	164 (65.1%)
If you're overweight, you're two to six times more likely to develop HBP.	True	134 (53.2%)
Regular exercise can help reduce BP.	True	147(58.3%)
Drinking alcohol lowers BP.	False	172 (68.3%)
HBP is a man's problem.	False	152 (60.3%)
Pregnancy-related HBP is temporary and doesn't require follow-up after delivery.	False	168 (66.7%)
BP gets lower in cold weather.	False	146 (57.9%)
Pakistanis eat 2 to 3 times more salt or sodium than they need.	True	126(50.0%)

Stratification of data was done with regard to the HKT score, as depicted in Table 3, to show a significant statistical association of good hypertension knowledge (HKT score = 9-12) with younger age (p-value <0.001), being unmarried (p-value = 0.001), being educated (p-value <0.001), being employed (p-value <0.001), duration of hypertension ≤5 years (p-value = 0.039), absence of ischemic heart disease (p-value = 0.002), absence of chronic kidney disease (p-value = 0.024), and having a positive family history of hypertension (p-value <0.001). No statistical association was seen with gender (p-value = 0.336) or diabetes mellitus (p-value = 0.053).

**Table 3:** Stratification of data with regard to the HKT score (n = 252) *p-value ≤0.05 is considered significant. HKT: hypertension knowledge test.

Demographic and clinical variables		Hypertension knowledge according to the HKT score, n(%)		p-value
		Poor (HKT score 0-8)	Good (HKT score 9-12)	
Age	≤44 years	82 (68.9%)	37 (31.1%)	<0.001*
	≥45 years	121(91.0%)	12 (9.0%)	
Gender	Female	127 (82.5%)	27 (17.5%)	0.336
	Male	76 (77.6%)	22 (22.4%)	
Marital status	Married	170 (84.6%)	31 (15.4%)	0.001*
	Unmarried	33 (64.7%)	18 (35.3%)	
Educational status	≤5 years	132 (97.1%)	04 (2.9%)	<0.001*
	6-12 years	67 (76.1%)	21 (23.9%)	
	≥13 years	04 (14.3%)	24 (85.7%)	
Employment status	Employed	65 (65.0%)	35 (35.0%)	<0.001*
	Unemployed	138 (90.8%)	14 (9.2%)	
Duration of disease (hypertension)	≤5 years	79 (74.5%)	27 (25.5%)	0.039*
	≥6 years	124 (84.9%)	22 (15.1%)	
Diabetes mellitus	Present	114 (85.1%)	20 (14.9%)	0.053
	Absent	89 (75.4%)	29 (24.6%)	
Ischemic heart disease	Present	85 (90.4%)	09 (9.6%)	0.002*
	Absent	118 (74.7%)	40 (25.3%)	
Chronic kidney disease	Present	27 (96.4%)	01 (3.6%)	0.024*
	Absent	176 (78.6%)	48 (21.4%)	
Family history of hypertension	Present	133 (74.3%)	46 (25.7%)	<0.001*
	Absent	70 (95.9%)	03 (4.1%)	

Regarding medication adherence, 26 (10.3%) forget to take their medication all the time, while 65 (25.8%) do so most of the time, as depicted in Table 4. Additionally, 17 (6.7%) have decided not to take their medication, and 48 (19.0%) have done so most of the time. In terms of dietary habits, 15 (6.0%) eat salty foods all the time, and 60 (23.8%) eat them most of the time, while 10 (4.0%) always add salt to their food before eating, and 52 (20.6%) do so most of the time, as demonstrated in Table 4. Regarding fast food consumption, 21 (8.3%) eat it all the time, and 49 (19.4%) do so most of the time. When it comes to healthcare management, 68 (27.0%) always make their next doctor’s appointment before leaving the office, while 99 (39.3%) do so most of the time. However, 21 (8.3%) have missed a scheduled appointment, and 54 (21.4%) do so most of the time. As for prescription refills, 22 (8.7%) forget to get their prescriptions filled, and 43 (17.1%) do so most of the time. Additionally, 17 (6.7%) have run out of their blood pressure medication, and 43 (17.1%) have done so most of the time, as depicted in Table 4. Finally, 17 (6.7%) always skip their medication before going to the doctor, and 43 (17.1%) do so most of the time.

**Table 4:** Practices toward hypertension control and treatment adherence (n = 252)

Clinical questions	Response, n(%)			
	All the time	Most of the time	Sometimes	Never
Do you forget to take your high blood pressure medicine?	26 (10.3%)	65 (25.8%)	109 (43.3%)	52 (20.6%)
Have you decided not to take your high blood pressure medication?	17 (6.7%)	48 (19.0%)	99 (39.3%)	88 (34.9%)
How often do you eat salty foods?	15 (6.0%)	60 (23.8%)	126 (50.0%)	51 (20.2%)
Do you add salt to your food before eating?	10 (4.0%)	52 (20.6%)	104 (41.3%)	86 (34.1%)
How often do you eat fast food?	21 (8.3%)	49 (19.4%)	93 (36.9%)	89 (35.3%)
Do you make your next doctor’s appointment before leaving the office?	68 (27.0%)	99 (39.3%)	61 (24.2%)	24 (9.5%)
Have you missed a scheduled appointment?	21 (8.3%)	54 (21.4%)	113 (44.8%)	64 (25.4%)
Do you forget to get your prescription filled?	22 (8.7%)	43 (17.1%)	124 (49.2%)	63 (25.0%)
Have you run out of your high blood pressure pills?	17 (6.7%)	43 (17.1%)	114 (45.2%)	78 (31.0%)
Do you skip your high blood pressure medication before going to the doctor?	17 (6.7%)	43 (17.1%)	94 (37.3%)	98 (38.9%)

## Discussion

The majority of patients in our study had poor knowledge about hypertension, which aligns with previous studies that emphasize the lack of awareness among hypertensive patients regarding the severity and management of the disease [11,12]. This underscores the need for improved educational initiatives to enhance patient understanding and promote better self-management of hypertension. Patients’ understanding of hypertension’s risk factors, symptoms, and complications could be improved, especially in rural or underprivileged settings where educational campaigns may not be widespread [10,13]. Targeted outreach and community-based education programs could help raise awareness and foster better management of the condition in these populations. Our findings regarding adherence to antihypertensive medication and lifestyle modifications, such as diet and exercise, align with previous studies that highlight common challenges in maintaining consistent treatment regimens, including forgetfulness, lack of motivation, and difficulty making long-term lifestyle changes [14,15]. These challenges are frequently observed across diverse patient populations and underscore the need for tailored strategies to improve adherence.

The high percentage of patients who forget or decide not to take medication in our study underlines the importance of improved medication reminders, better communication between healthcare providers and patients, and the role of family support [10,16]. This behavior may stem from a lack of understanding about the importance of consistent adherence, side effects such as dizziness or fatigue, and financial constraints that make medications less accessible. Additionally, insufficient follow-up care and support from healthcare providers can lead to reduced motivation for maintaining treatment regimens. The association between good knowledge of hypertension and factors such as younger age, marital status, education, and absence of comorbid conditions is valuable. This suggests that interventions targeting younger, unmarried, and less educated individuals could be prioritized to improve knowledge and adherence. Since younger individuals demonstrated better hypertension knowledge in this study, the recommendation to target younger, unmarried, and less educated individuals primarily stems from their challenges with medication adherence rather than knowledge gaps. While their understanding of hypertension may be stronger, factors such as lifestyle habits, medication side effects, and lack of consistent follow-up care may contribute to difficulties in maintaining adherence, making them a key group for intervention.

The present study offers valuable insights into hypertension knowledge and treatment adherence in a specific cohort of patients in Lahore, Pakistan. However, several limitations need to be considered. The cross-sectional design limits the ability to establish causal relationships, as it captures data at a single point in time, making it difficult to assess the long-term effects of hypertension knowledge and adherence on health outcomes. Additionally, the reliance on self-reported data for lifestyle habits introduces recall bias, as participants may misreport or overstate behaviors such as medication adherence or dietary practices, which could skew the findings. The use of a single-site sample further restricts the generalizability of the results to other populations or healthcare settings, as regional differences in healthcare infrastructure and cultural attitudes may influence outcomes. These factors collectively affect the external validity and depth of the study’s conclusions.

Future research should aim to address these limitations through longitudinal designs, more objective measures of adherence, and multi-center studies that cover diverse geographic regions. A broader scope would help validate the findings and guide the development of more effective, evidence-based interventions for hypertension management.

Based on the findings of this study, several recommendations can be made to improve hypertension knowledge and adherence among patients. First, targeted educational programs should focus on raising awareness about hypertension, especially among younger, unmarried, and less educated individuals. Healthcare providers should deliver clear, culturally sensitive information and promote regular medication adherence through the use of reminder systems. Healthier lifestyles, including reduced salt intake and increased physical activity, should also be encouraged [14,16]. Family involvement in the educational process can create a supportive environment for patients [10,15]. Regular follow-ups and blood pressure monitoring remain essential components of effective management. Future research should explore the impact of specific interventions on improving hypertension care, including the use of community-based programs, digital health tools, and policy-level strategies for broader public health implementation.

Educational programs should be delivered through a combination of clinics, community workshops, and media campaigns to ensure broad accessibility. Clinics provide personalized education during patient visits, while community workshops and media campaigns (such as radio, TV, or social media) can raise awareness, especially in rural or underserved areas. To improve medication adherence, reminder systems like phone calls, SMS reminders, or mobile apps can be highly effective in prompting patients to take their medications on time and attend follow-up appointments. Mobile apps can also track medication schedules, send notifications, and offer educational resources. Family involvement can be structured through home-based counseling, where healthcare providers educate both the patient and their family members. Caregiver training can help families understand the importance of adherence, recognize side effects, and provide emotional support, creating a supportive environment that encourages better adherence and healthier lifestyle choices. To promote lifestyle changes like salt reduction and exercise, policy-level strategies such as food labeling regulations and workplace wellness programs can be effective. Regular follow-ups could be enhanced with intervention models like pharmacist-led blood pressure monitoring or telemedicine, offering remote consultations and support. Future research should focus on assessing the effectiveness of mobile health interventions for medication adherence, the impact of telemedicine on hypertension management, and the role of policy interventions like food labeling in encouraging healthier behaviors.

## Conclusions

This study underscores the significant gap in hypertension knowledge and the suboptimal adherence to treatment among hypertensive patients in Lahore, Pakistan. The findings highlight the need for comprehensive educational programs and improved strategies to promote treatment adherence, and the importance of focusing not only on younger patients but also on older and less educated individuals, who exhibited poorer knowledge about hypertension. Given the high prevalence of comorbidities such as diabetes and ischemic heart disease in this cohort, these conditions complicate hypertension management by increasing the medication burden and introducing lifestyle constraints. Patients with these comorbidities often require multiple medications, which can lead to confusion, missed doses, and poor adherence, while their conditions may necessitate dietary restrictions and more frequent healthcare visits, making sustained hypertension control challenging.

Future research should focus on developing tailored interventions to improve both knowledge and adherence in diverse patient populations. Effective interventions could include community health worker programs to offer tailored support, digital health tools like mobile apps for monitoring and reminders, and pharmacist-led adherence programs to ensure patients understand their treatment regimens. Policy-level approaches, such as integrating hypertension education into primary care visits and creating national guidelines for managing comorbid hypertension, could also play a crucial role in improving outcomes. Long-term follow-up studies or intervention trials are essential to assess whether improvements in knowledge and adherence lead to better blood pressure control and, ultimately, better cardiovascular outcomes.

## Appendices

The structured questionnaire, consisting of three domains, used in the present study is as follows: (1) Domain 1, clinical and demographic information (Table 5); (2) Domain 2, hypertension knowledge test (Table 6); and (3) Domain 3, practices toward hypertension control and treatment adherence(Table 7).

**Table 5:** Demographic and clinical information

Variables	Responses
Age in years	
Gender	Female
	Male
Marital status	Married
	Unmarried
Educational status	≤5 years
	6-12 years
	≥13 years
Employment status	Employed
	Unemployed
Duration of disease (hypertension) in years	
Diabetes mellitus	Present
	Absent
Ischemic heart disease	Present
	Absent
Chronic kidney disease	Present
	Absent
Family history of hypertension	Present
	Absent

**Table 6:** Hypertension knowledge test (HKT)

HKT statement	Options	
If your mother or father has high blood pressure (HBP), your chance of getting it is higher.	True	False
Young adults don't get HBP.	True	False
HBP always has symptoms.	True	False
HBP is not life-threatening.	True	False
Blood Pressure (BP) is high when it's at or over 140/90 mmHg.	True	False
If you're overweight, you're two to six times more likely to develop HBP.	True	False
Regular exercise can help in reducing BP.	True	False
Drinking alcohol lowers BP.	True	False
HBP is a men's problem.	True	False
Pregnancy related HBP is temporary and doesn't require follow-up after delivery.	True	False
BP gets lower in cold weather.	True	False
Pakistanis eat 2 to 3 times more salt or sodium than they need.	True	False

**Table 7:** Practices toward hypertension control and treatment adherence

Questions	Options			
Do you forget to take your high blood pressure medicine?	All the time	Most of the time	Sometimes	Never
Have you decided not to take your high blood pressure medication?	All the time	Most of the time	Sometimes	Never
How often do you eat salty foods?	All the time	Most of the time	Sometimes	Never
Do you add salt to your food before eating?	All the time	Most of the time	Sometimes	Never
How often do you eat fast food?	All the time	Most of the time	Sometimes	Never
Do you make your next doctor’s appointment before leaving the office?	All the time	Most of the time	Sometimes	Never
Have you missed a scheduled appointment?	All the time	Most of the time	Sometimes	Never
Do you forget to get your prescription filled?	All the time	Most of the time	Sometimes	Never
Have you run out of your high blood pressure pills?	All the time	Most of the time	Sometimes	Never
Do you skip your high blood pressure medication before going to the doctor?	All the time	Most of the time	Sometimes	Never

## References

[1] Schutte AE, Srinivasapura Venkateshmurthy N, Mohan S, Prabhakaran D (2021). Hypertension in low- and middle-income countries. Circ Res.

[2] Citoni B, Figliuzzi I, Presta V (2022). Prevalence and clinical characteristics of isolated systolic hypertension in young: analysis of 24 h ambulatory blood pressure monitoring database. J Hum Hypertens.

[3] Hossain MB, Parvez M, Islam MR, Evans H, Mistry SK (2022). Assessment of non-communicable disease related lifestyle risk factors among adult population in Bangladesh. J Biosoc Sci.

[4] Muli S, Meisinger C, Heier M, Thorand B, Peters A, Amann U (2020). Prevalence, awareness, treatment, and control of hypertension in older people: results from the population-based KORA-age 1 study. BMC Public Health.

[5] Parati G, Lombardi C, Pengo M, Bilo G, Ochoa JE (2021). Current challenges for hypertension management: from better hypertension diagnosis to improved patients' adherence and blood pressure control. Int J Cardiol.

[6] Hamrahian SM, Maarouf OH, Fülöp T (2022). A critical review of medication adherence in hypertension: barriers and facilitators clinicians should consider. Patient Prefer Adherence.

[7] Butt NI, Ghoauri MSA, Sabeh D (2024). Prevalence of hypertension and its association with diabetic complications in patients with type 2 diabetes mellitus at a tertiary care hospital. J Med Res Rev.

[8] Wei X, Khan N, Durrani H (2023). Protocol for a pragmatic cluster randomised controlled trial to evaluate the effectiveness of digital health interventions in improving non-communicable disease management during the pandemic in rural Pakistan. PLoS One.

[9] Han HR, Chan K, Song H, Nguyen T, Lee JE, Kim MT (2011). Development and evaluation of a hypertension knowledge test for Korean hypertensive patients. J Clin Hypertens (Greenwich).

[10] Daniel A, Saddique H, Tasneem SS (2024). The assessment of knowledge regarding hypertension and treatment adherence. J Health Rehabil Res.

[11] Dehghan M, Dehghan Nayeri N (2021). Factors affecting hypertensive treatment adherence: development and preliminary validation of a new scale. Int J Hypertens.

[12] Pristianty L, Hingis ES, Priyandani Y, Rahem A (2023). Relationship between knowledge and adherence to hypertension treatment. J Public Health Afr.

[13] Jankowska-Polańska B, Uchmanowicz I, Dudek K, Mazur G (2016). Relationship between patients' knowledge and medication adherence among patients with hypertension. Patient Prefer Adherence.

[14] Marseille BR, Commodore-Mensah Y, Davidson PM, Baker D, D'Aoust R, Baptiste DL (2021). Improving hypertension knowledge, medication adherence, and blood pressure control: a feasibility study. J Clin Nurs.

[15] Ayodapo AO, Elegbede OT, Omosanya OE, Monsudi KF (2020). Patient education and medication adherence among hypertensives in a tertiary hospital, south western Nigeria. Ethiop J Health Sci.

[16] Delavar F, Pashaeypoor S, Negarandeh R (2020). The effects of self-management education tailored to health literacy on medication adherence and blood pressure control among elderly people with primary hypertension: a randomized controlled trial. Patient Educ Couns.

